# Continuing Professional Development—Medical Imaging

**DOI:** 10.1002/jmrs.70057

**Published:** 2026-01-09

Maximise your continuing professional development (CPD) by reading the selected article and answering the five questions. Please remember to self‐claim your CPD and retain your supporting evidence. Answers will be available via the QR code and published in JMRS—Volume 73, Issue 4, December 2026.

## An Analysis of Acquisition Dose in Male Mammography

Zechary Ng, Maeve Masterson, Daniel Carrion, Mohamed K. Badawy, https://doi.org/10.1002/jmrs.70027.

1. Which factor primarily motivated the authors to investigate acquisition dose in male mammography in this study?
  - Increasing male breast cancer incidence requiring imaging optimisation.
  - Risk of underdiagnosis related to low male breast tissue density.
  - Limited availability of published acquisition dose data for male mammography.
  - Differences in positioning techniques between male and female patients.
2. Which statement best explains the difference in mean acquisition dose observed between full‐field digital mammography (FFDM) and digital breast tomosynthesis (DBT) observed in this study?
  - DBT delivers a higher dose because multiple low‐dose projections are acquired and summed.
  - FFDM delivers a higher dose because it uses a single high‐dose exposure.
  - DBT delivers a lower dose because each projection uses reduced mAs.
  - No meaningful dose difference exists because male breast thickness is minimal.
3. According to the study's analysis, which variable demonstrated the strongest influence on acquisition dose variation in male mammography?
  - kVp
  - mAs
  - Compressed breast thickness
  - Imaging system model
4. When comparing acquisition doses between male and female mammography, which methodological limitation most affected interpretation of the results?
  - Operator‐dependent variation in radiographer technique.
  - Differences in breast thickness and tissue composition between sexes.
  - Inconsistent positioning methods used for male and female patients.
  - Lack of dose calibration between FFDM and DBT systems.
5. What is the most appropriate clinical implication arising from the study's findings?
  - Exposure parameters should be optimised with consideration of male breast anatomy.
  - Routine DBT screening should replace FFDM for all male patients.
  - Radiation dose protocols should be identical for males and females.
  - Mammography is unsuitable for assessment of male breast disease.

## Answers

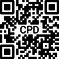

Scan this QR code to find the answers.
